# COVID-19 Vaccinations: Perceptions and Behaviours in People with Primary Ciliary Dyskinesia

**DOI:** 10.3390/vaccines9121496

**Published:** 2021-12-17

**Authors:** Eva S. L. Pedersen, Maria Christina Mallet, Yin Ting Lam, Sara Bellu, Isabelle Cizeau, Fiona Copeland, Trini Lopez Fernandez, Michele Manion, Amanda L. Harris, Jane S. Lucas, Francesca Santamaria, Myrofora Goutaki, Claudia E. Kuehni, COVID-PCD Patient Advisory Group

**Affiliations:** 1 Institute of Social and Preventive Medicine, University of Bern, 3012 Bern, Switzerland; eva.pedersen@ispm.unibe.ch (E.S.L.P.); maria.mallet@ispm.unibe.ch (M.C.M.); yin.lam@ispm.unibe.ch (Y.T.L.); myrofora.goutaki@ispm.unibe.ch (M.G.); 2 Graduate School for Health Sciences, University of Bern, 3012 Bern, Switzerland; 3 Associazione Italiana Discinesia Ciliare Primaria Sindrome di Kartagener Onlus, 70124 Bari, Italy; saradcp@virgilio.it; 4 Association ADCP, 42218 Saint-Étienne, France; icizeau@cegetel.net; 5 PCD Support UK, London MK18 9DX, UK; fiona.copeland@stonac.co.uk; 6 Asociación Española de Pacientes con Discinesia Ciliar Primaria, Santo Ángel 30151, Philippines; asociaciondcpes@gmail.com; 7 PCD Foundation, Minneapolis, MN 55420, USA; michelemanion@gmail.com; 8 Primary Ciliary Dyskinesia Centre, NIHR Biomedical Research Centre, University Hospital Southampton NHS Foundation Trust, Southampton SO16 6YD, UK; Amanda-lea.harris@uhs.nhs.uk (A.L.H.); jlucas1@soton.ac.uk (J.S.L.); 9 Faculty of Medicine, School of Clinical and Experimental Medicine, University of Southampton, Southampton SO17 1BJ, UK; 10 Department of Translational Medical Sciences, Federico II University, 80138 Naples, Italy; santamar@unina.it; 11 Division of Paediatric Respiratory Medicine and Allergology, Department of Paediatrics, Inselspital, Bern University Hospital, University of Bern, 3010 Bern, Switzerland

**Keywords:** SARS-CoV-2, COVID-19, PCD, primary ciliary dyskinesia, vaccine, vaccinations, pandemic

## Abstract

Primary ciliary dyskinesia (PCD) is a rare genetic disease that causes recurrent respiratory infections. People with PCD may be at higher risk of severe coronavirus disease 2019 (COVID-19), and therefore vaccination against severe acute respiratory syndrome coronavirus 2 (SARS-CoV-2) is important. We studied vaccination willingness, speed of vaccination uptake, side effects, and changes in social contact behaviour after vaccination in people with PCD. We used data from COVID-PCD, an international participatory cohort study. A COVID-19 vaccination questionnaire was emailed to participants in May 2021 and 423 participants from 31 countries replied (median age: 30 years, range 1–85 years; 261 (62%) female). Vaccination uptake and willingness were high, with 273 of 287 adults (96%) being vaccinated or willing to be in June 2021; only 4% were hesitant. The most common reason for hesitancy was fear of side effects, reported by 88%. Mild side effects were common, but no participant reported severe side effects. Half of the participants changed their social behaviour after vaccination by seeing friends and family more often. The high vaccination willingness in the study population might reflect the extraordinary effort taken by PCD support groups to inform people about COVID-19 vaccination. Clear and specific information and involvement of representatives is important for high vaccine uptake.

## 1. Introduction

Vaccination against coronavirus disease 2019 (COVID-19) has proven effective in preventing transmission of severe acute respiratory syndrome coronavirus 2 (SARS-CoV-2) [1,2] with most countries now vaccinating against COVID-19 [3]. Several vaccines against COVID-19 have been approved [4,5,6], the first of which were administered at the end of 2020. Priority was given to people considered at high risk of severe COVID-19, such as the elderly and people with chronic diseases [7]. By August 2021, many European countries had vaccinated most of the people willing to get vaccinated. Vaccination willingness has generally been high in Europe, but an important proportion of the general population in several countries still hesitates to get vaccinated [8,9,10]. In some countries, vaccine uptake has only reached 50% of the general population [11].

There are few data about vaccination hesitancy among people who are at high risk of severe COVID-19, particularly in populations with rare diseases such as primary ciliary dyskinesia (PCD). PCD is a rare genetic multi-system disease where dysfunctional cilia lead to impaired mucociliary clearance, situs defects, congenital heart defects, and other health problems [12,13,14,15,16]. PCD is characterized by chronic upper and lower airway disease [13,17,18,19,20], reduced lung function, and in some cases supplementary oxygen [21,22,23,24,25]. At the beginning of the pandemic, people with PCD and other chronic respiratory diseases were thought to be at high risk of severe COVID-19 disease. To study the incidence and severity of COVID-19 in people with PCD, we set up a participatory study, the COVID-PCD, in collaboration with PCD support groups from all over the world [26]. First results from the study showed a low infection rate and mostly mild COVID-19 disease [27]. People with PCD were strongly recommended to get vaccinated against SARS-CoV-2; however, to date there is no information about vaccination willingness, vaccination uptake, and side effects among people with PCD. Studies including people with other pre-existing health conditions show varying vaccination willingness [28,29,30]. The speed of vaccination rollout differs between countries, and it is unclear how quickly high-risk populations are being vaccinated. The aim of this study was to describe COVID-19 vaccination willingness and hesitancy among people with PCD, to study the speed of vaccination uptake, assess the vaccines’ reported side effects, and investigate changes in social contact behaviour after vaccination.

## 2. Materials and Methods

### 2.1. Study Design and Inclusion Criteria

We used data from COVID-PCD, an international prospective cohort study designed to follow people with PCD during the COVID-19 pandemic period (clinicaltrials.gov: NCT04602481). COVID-PCD is a participatory research project where people with PCD play an active role at all stages of research, including study design and content, piloting, and communication of results. Details about study methods and initial results have been published previously [26,27]. In summary, COVID-PCD includes people of any age from any country with suspected or confirmed PCD. Data are collected through anonymous online questionnaires that are adapted to three age groups; children below 14 years, adolescents between 14 and 17 years, and adults aged 18 years or older. Parents complete the questionnaires for children below 14 years, although the children are encouraged to participate. Adolescents and adults complete questionnaires themselves. The study is available in five languages, including English, German, Spanish, Italian, and French. Recruitment started on 31 May 2020. The Cantonal Ethics Committee of Bern approved the study (Study ID: 2020-00830). Participants provide consent to participate online at the time of registration to the study. This article follows the STROBE reporting recommendations [31].

### 2.2. Study Procedures

PCD support groups helped recruit study participants by contacting people living with PCD through social media and email networks and encouraged them to take part in the study. Participants register in the COVID-PCD study through a link on the study website (Available online: www.covid19pcd.ispm.ch, accessed on 29 May 2021). At registration, participants read detailed information about the study and provide consent online. Participants first complete a baseline questionnaire with questions about their disease, symptoms, and SARS-CoV-2 infections experienced prior to joining the study [32]. Thereafter they receive a short follow-up questionnaire each week with questions on incident SARS-CoV-2 infections, COVID-19 vaccinations, current symptoms, and social contact behaviour. Intermittently, participants receive short special questionnaires that focus on specific topics. This paper presents data from a special questionnaire on vaccinations that was sent to participants on 29 May 2021 as well as data on vaccinations retrieved through the weekly questionnaire. Participants received up to two reminders if they did not respond to the special questionnaire. Participating PCD support groups were strongly involved in the development of all questionnaires. All data were entered in a Research Electronic Data Capture (REDCap) database (Available online: www.project-redcap.org/ (accessed on 15 December 2021)) [33,34], which is hosted by the Swiss medical registries and data linkage centre (SwissRDL) at the University of Bern, Switzerland.

### 2.3. Information about COVID-19 Vaccinations

The special questionnaire on COVID-19 vaccinations asked whether participants had already been vaccinated, and if yes, which vaccine they received (Supplementary Table S1). We also asked whether participants changed their social contact behaviour after vaccination. Unvaccinated participants were asked about vaccination willingness. We distinguished between people who planned to get vaccinated, were not sure whether to get vaccinated, or did not want to get vaccinated. Additional questions focused on reasons for and against vaccination.

The routine weekly questionnaire asked whether participants had been vaccinated since completion of the previous weekly questionnaire, which date they were vaccinated, whether it was the first or the second vaccination, and which side effects they experienced. We asked about side effects separately after the first and the second vaccine.

### 2.4. Statistical Analyses

We described the demographics of the study participants, vaccination uptake, vaccination willingness and hesitancy, reasons for and against getting vaccinated, side effects, changes in social behaviour using number and proportion for categorical variables, and mean and standard deviation (SD) or median and interquartile range for continuous variables. We described vaccination willingness and hesitancy per age group. We chose the cut-off of 12 years between the child and adolescent age groups because several vaccines had been approved for children down to the age of 12 years by June 2021. We described speed of vaccination uptake using the date of the first vaccination extracted from the weekly follow-up questionnaires and used this to compare how quickly participants living in different countries were vaccinated. To study factors associated with reported side effects we used multilevel logistic regression. We ran a regression with each side effect as a dependent variable (no side effects, local pain or swelling around injection site, tiredness, fever, muscle or bone pain, other) and added the following explanatory variables: age, sex, vaccine type, and vaccine timing (first or second injection, included as a separate level representing repeated measurements within an individual). The logistic regression analyses are reported as odds ratios (ORs) and 95% confidence intervals (95% CIs). Our dataset had less than 2% missing values of single variables and records with missing data were excluded from the analysis. We used STATA version 15 for statistical analysis.

## 3. Results

In total, 423 of the 689 study participants (61%) completed the special questionnaire on vaccinations. The median age was 30 years (age range: 1–85 years, interquartile range 12 to 47) and 261 (62%) were female (Supplementary Table S2). Study participants were from 31 different countries with the highest numbers from the UK (*n* = 88; 21%), Germany (*n* = 76; 18%), USA (*n* = 64; 15%), and Switzerland (*n* = 31; 7%). Participants who completed the special questionnaire on vaccinations were slightly older, more often female, and more often from European countries than those who did not complete it (Supplementary Table S2).

Vaccination uptake and willingness were high in the study population. Among the 287 adults, 263 (92%) had already been vaccinated, 3 (1%) had an appointment, 7 (2%) planned to get vaccinated, 7 (2%) were hesitant to get vaccinated, and 7 (2%) did not plan to get vaccinated (Table 1). People who were unsure or unwilling to get vaccinated more often came from Germany, Switzerland, and other European countries, but we found no difference in age or sex (Supplementary Table S3). Among the 41 adolescent study participants, 17 (41%) had already been vaccinated, and among children below 12 years, 2 of 95 (2%) were vaccinated. Five adolescents (12%) and 8 children (8%) did not plan to get vaccinated, even if the vaccine were to be approved for persons their age. Among participants who had been vaccinated, most had received Pfizer-BioNTech (48%) followed by AstraZeneca (22%), Moderna (15%), and other (15%).

**Figure 1:**
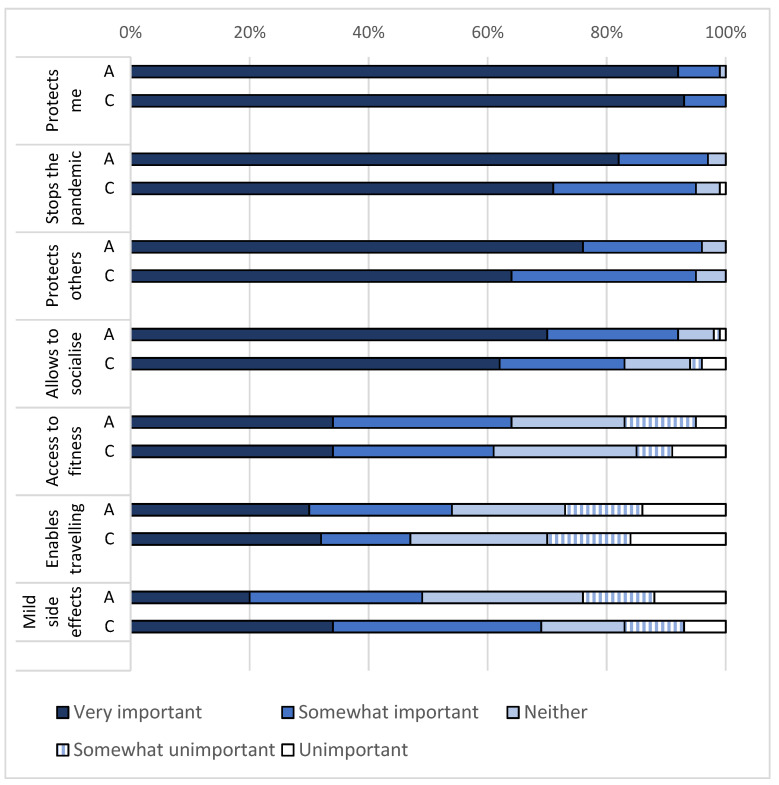
Reasons for getting vaccinated among participants who already got vaccinated or wanted to get vaccinated among adults (*n* = 272) and children and adolescents (*n* = 94). Responses rated from very important to unimportant. (COVID-PCD study, May 2021). Abbreviations: A = adults aged 18 years or above; C = children and adolescents below 18 years.

**Figure 2:**
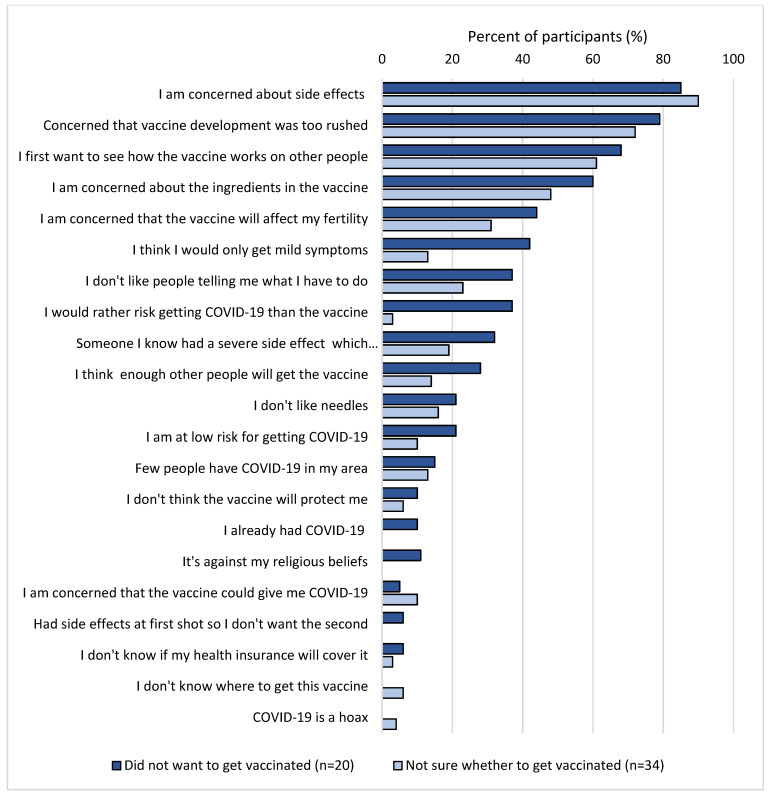
Agreement (rated as somewhat or strongly) with reasons against getting vaccinated among those who did not want to get vaccinated against COVID-19 (*n* = 20) and those who were not sure whether to get vaccinated (*n* = 34). (COVID-PCD study, May 2021).

The most important reasons for getting vaccinated were to “protect themselves and others from infection” (99%) and to “stop the pandemic” (95%) (Figure 1). Less important reasons to get vaccinated were that the vaccination would enable them to travel and would make activities such as going to the fitness centre possible. Reasons against getting vaccinated were similar among those who did not want to get vaccinated (*n* = 20) and those who were not sure whether to get vaccinated (*n* = 31) (Figure 2). In total, 88% of those who were hesitant to get vaccinated reported that they were concerned about side effects and 75% reported concern that the vaccine development had been too rushed. Few participants reported reasons relating to disbelief in the effectiveness of vaccines (8%) or not knowing how to get the vaccine (4%).

The vaccination uptake and the speed of vaccination rollout differed between countries (Supplementary Figures S1 and S2). Participants living in the UK were vaccinated faster than in all other countries. By mid-February 2021, 80% of the adults who had completed the special vaccination questionnaire had received at least one vaccine and by the end of June 2021, 100% had received at least one vaccine. The second fastest country to vaccinate was the USA, followed by Switzerland. The country in which the vaccination rollout was slowest was Australia, where by June 2021, 80% of participants had received at least one vaccine.

**Figure 3:**
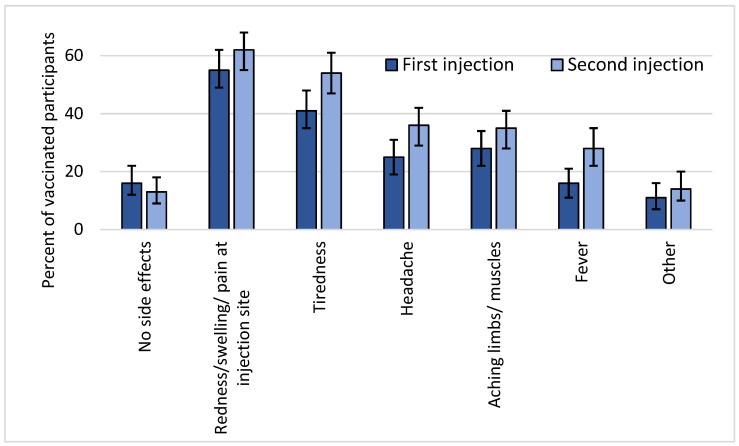
Self-reported side effects after first (*n* = 238) and after second vaccine (*n* = 214) among vaccinated participants including 95% confidence intervals (COVID-PCD study, May 2021). Other side effects included nausea, vomiting, diarrhoea, stomachache, dizziness, chills, breathlessness, cough, congestion, and swollen lymph nodes.

Reported side effects after vaccination were common but mild; nobody reported severe side effects. The most common side effect was redness, swelling, or pain around the injection site, which was reported by 60% of participants, followed by tiredness, headache, aching muscles, and fever (Figure 3). Other side effects such as nausea, vomiting, diarrhoea, and stomachache were rare. Participants reported side effects more often after the second vaccine than the first. Younger participants more often reported fever, headache, and muscle pain than older participants (Table 2). Participants who received Moderna or AstraZeneca reported side effects more often than participants who received Pfizer-BioNTech.

**Figure 4:**
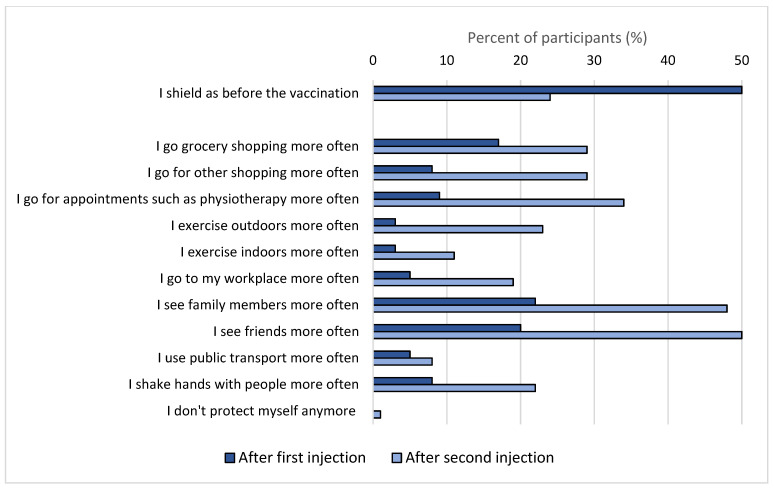
Proportion of participants who reported how they changed their social behaviour after first and second vaccinations compared to before getting vaccinated (*n* = 282) (COVID-PCD study, May 2021).

Half of the participants changed their social behaviour after the first vaccine, and three quarters after the second vaccine (Figure 4). After the first vaccine, participants started to go grocery shopping more often (reported by 23%) as well as to meet family and friends (20%). After the second vaccine, there was an increase in the frequency of participants seeing family and friends (50%), attending appointments such as physiotherapy (34%), and going shopping (29%).

## 4. Discussion

This international participatory study found that most people with PCD were already vaccinated against COVID-19 or willing to be by the end of May 2021. Only 2% of adults did not plan to get vaccinated, for which the most common reasons were concerns about side effects and concerns that the vaccines had been developed too quickly. Side effects were common but mild, and were reported more often after the second vaccine than the first, more often by younger participants than older, and more often among participants vaccinated with Moderna or AstraZeneca than with Pfizer-BioNTech. Half of the participants changed their social behaviour after the first vaccine, with the main difference being an increase in time spent with family and friends.

### 4.1. Interpretation and Comparison with Other Studies

Vaccination willingness in our study population was high, with 96% of adults and 73% of adolescents aged 12–17 years being vaccinated or willing to be by June 2021. Two children below the age of 12 years had been vaccinated and 69% of parents reported that they were willing to get their child vaccinated if the vaccine were to be approved for children. Few studies are published on COVID-19 vaccination uptake and willingness with comparison across different countries. Most of these data stem from convenience sampling, which has a high risk of selection bias [35]. One study from the UK included more than two million unselected adults and found a vaccination willingness of 94% [36], similar to what we observed in our study population. However, other studies show lower vaccination willingness in the general population. A study from the UK showed a vaccination willingness of 75%, with willingness being higher among study participants born in the UK compared to other countries of birth [37]. In a study from the USA including more than 75,000 adults, 80% of the study participants had been vaccinated or were willing to be by the end of March 2021 [38]. In our study, we found that unwillingness to get vaccinated was more commonly reported in countries such as Switzerland and Germany than in countries such as the UK (Supplementary Table S3). When looking at whole-country vaccination rates from Our World in Data [11], overall vaccination rates were also higher in the UK than for example Switzerland. Few studies have reported data on vaccination willingness and uptake in people with chronic respiratory disease. A study in 273 adults with severe asthma showed high willingness to get vaccinated, with only 6% refusing vaccination, which is in line with our findings [30]. In contrast with our findings, only 20% reported side effects after the first and second doses of vaccine. A study from India among adults with diabetes found a vaccination willingness of 64% [28]. Reasons for not getting vaccinated included fear of side effects, not knowing about the vaccine, and needing to discuss with family members. In our study, nobody reported not knowing about the vaccine. PCD support groups around the world have actively informed members about COVID-19 vaccines and encouraged people with PCD to get vaccinated. This information and encouragement may have contributed to the vaccination uptake and willingness in our population.

We found that reported side effects were common but mild, which has also been observed in the general population. In a large prospective cohort study in more than half a million adults from the UK, local side effects such as pain, swelling, and redness around the injection site were reported by 72% after the first dose of Pfizer-BioNTech and by 59% after the first dose of AstraZeneca [39]. Systemic side effects such as headache, fatigue, chills, and fever were reported by 14% after a dose of Pfizer-BioNTech and by 34% after AstraZeneca. Similar findings were seen in two studies among healthcare workers [40,41]. In our population, all side effects were more often reported after the second injection than after the first. We found that systemic side effects were more often reported by participants who received Moderna or AstraZeneca than by people who received Pfizer-BioNTech, which is in line with what was found in the UK study [39]. Younger persons were more likely to report side effects than older persons, which has also been shown in other studies and may be explained by a stronger immune response to the vaccine in younger people [42]. Other studies reported that women were more likely to experience side effects [39,41], although we did not observe this. In summary, our study does not suggest that people with PCD have more or worse side effects from the COVID-19 vaccines than the general population.

We found that half of the study participants changed their social behaviour after receiving the first dose of the vaccine. Participants started to see family and friends more often, but only very few (1% of participants) reported not using additional protective measures after vaccination. There are few published data from the general population on changes in social behaviour after vaccination but in a UK study among 23,287 adults, the authors found no evidence that people stopped social distancing after vaccination [43]. In our study, we did not ask whether participants kept social distancing when going out or seeing friends and family, but around 20% said that they did shake hands with people more often after getting vaccinated. This is in contrast to previous studies that found that people with PCD are very careful in protecting themselves against COVID-19 by avoiding public places and always wearing facemasks [44].

### 4.2. Strengths and Limitations

A major strength of this study is the large sample size of people with PCD from all over the world. PCD is a rare disease, and it can be difficult to recruit participants for research studies. COVID-PCD is a participatory study that was initiated, designed, and tested in collaboration with people who have PCD. This improved study participation. The PCD support groups advertised the special questionnaire on COVID-19 vaccinations via social media and email networks before it was sent out, which may have improved the response rate. The questionnaire was made available in five languages, which ensured that fewer people were excluded by language barriers. A limitation is that the study population may not be representative for all people with PCD but rather mainly of those who are in contact with a patient support group. It is difficult to ascertain the representativeness of the study population, as we have no information about people with PCD who did not participate in the COVID-PCD study. When we compare those who completed the special questionnaire on vaccinations (61%) to those who did not (41%), we see that non-responders were slightly younger than those who responded and tended to come from non-European countries (Supplementary Table S2). It is possible that respondents were more vaccine-willing than non-responders, which would have led to an overestimation of vaccine uptake. Another limitation is that the sample size, although large for a rare disease, had limited power to detect rare side effects of vaccination.

## 5. Conclusions

In summary, this study found that vaccination willingness and uptake was high among people with PCD, which may reflect the extraordinary effort made by PCD support groups to inform people with PCD about the advantages of vaccination. No severe side effects were reported. Clear and specific public information about COVID-19 vaccine safety is important for a high vaccine uptake, and thus for better protection against COVID-19.

## Figures and Tables

**Table 1:** Self-reported vaccination willingness, vaccination uptake, and vaccine type among people with primary ciliary dyskinesia by age (*n* = 423) (COVID-PCD study, May 2021).

Participants Who Completed the Vaccination Questionnaire (*n* = 423)	Adults Aged ≥ 18 y	Adolescents * Aged 12–17 y	Children * ≤ 11 y
	*n* = 287	*n* = 41	*n* = 95
	*n* (%)	*n* (%)	*n* (%)
Vaccination willingness			
Already vaccinated or willing to be vaccinated	273 (96)	30 (73)	66 (69)
Hesitant (not sure whether to get vaccinated)	7 (2)	6 (15)	21 (22)
Resistant (do not plan to get vaccinated)	7 (2)	5 (12)	8 (8)
Vaccinated against COVID-19			
Yes, I received 2 doses	198 (69)	7 (17)	1 (1)
Yes, I received 1 dose	65 (23)	10 (24)	1 (1)
No, but I have an appointment to get vaccinated	3 (1)	0	1 (1)
No	21 (7)	24 (59)	92 (97)
Participants who received 1 or 2 vaccine doses (*n* = 282)	*n* = 263	*n* = 17	*n* = 2
Type of vaccine			
Pfizer-BioNTech	120 (46)	13 (76)	1 (50)
Moderna	41 (16)	1 (6)	0
AstraZeneca	63 (24)	0	0
Janssen/Johnson and Johnson	3 (1)	0	0
I don’t know	36 (14)	3 (18)	1 (50)

* Data for children below 14 years of age were reported by their parents. Abbreviations: y, years.

**Table 2:** Odds ratios from multilevel logistic regression analyses of reporting side effects after COVID-19 vaccination adjusted for age, sex, first or second injection, and type of vaccine (*n* = 266) (COVID-PCD study, May 2021).

	OR	Lower 95% CI	Upper 95% CI	*p*-Value
**No side effects**				
Age (continuous, per year increase)	0.98	0.95	1.00	0.126
Sex (female vs. male)	1.80	0.72	4.50	0.208
Injection (second vs. first)	1.57	0.79	3.11	0.198
Vaccine (ref: Pfizer-BioNTech)				
Moderna	2.34	0.61	8.97	0.215
AstraZeneca	0.89	0.33	2.42	0.818
**Local pain/swelling**				
Age (continuous, per year increase)	0.99	0.96	1.01	0.320
Sex (female vs. male)	1.58	0.72	3.49	0.249
Injection (second vs. first)	1.45	0.86	2.45	0.168
Vaccine (ref: Pfizer-BioNTech)				
Moderna	3.32	1.11	9.92	0.032
AstraZeneca	0.54	0.22	1.30	0.167
**Tiredness**				
Age (continuous, per year increase)	0.99	0.97	1.01	0.374
Sex (female vs. male)	1.54	0.81	2.93	0.190
Injection (second vs. first)	2.19	1.33	3.61	0.002
Vaccine (ref: Pfizer-BioNTech)				
Moderna	2.95	1.23	7.09	0.016
AstraZeneca	1.12	0.55	2.29	0.751
**Fever**				
Age (continuous, per year increase)	0.97	0.95	0.99	0.015
Sex (female vs. male)	0.90	0.43	1.90	0.791
Injection (second vs. first)	3.12	1.64	5.94	0.001
Vaccine (ref: Pfizer-BioNTech)				
Moderna	5.64	2.06	15.42	0.001
AstraZeneca	7.57	2.93	19.53	<0.001
**Headache**				
Age (continuous, per year increase)	0.98	0.96	1.00	0.052
Sex (female vs. male)	1.73	0.83	3.62	0.147
Injection (second vs. first)	2.31	1.33	4.00	0.003
Vaccine (ref: Pfizer-BioNTech)				
Moderna	3.15	1.21	8.20	0.019
AstraZeneca	6.30	2.65	15.00	<0.001
**Muscle/bone pain (not around injection site)**				
Age (continuous, year increase)	0.97	0.95	0.99	0.003
Sex (female vs. male)	0.79	0.45	1.38	0.403
Injection (second vs. first)	1.59	0.99	2.56	0.057
Vaccine (ref: Pfizer-BioNTech)				
Moderna	2.34	1.11	4.96	0.026
AstraZeneca	4.73	2.34	9.53	<0.001
**Other**				
Age (continuous, per year increase)	1.01	0.97	1.04	0.751
Sex (female vs. male)	1.34	0.45	4.01	0.604
Injection (second vs. first)	0.68	0.78	3.61	0.183
Vaccine (ref: Pfizer-BioNTech)				
Moderna	3.93	0.95	16.19	0.058
AstraZeneca	4.22	1.24	14.38	0.021

## Data Availability

COVID-PCD data can be made available on reasonable request by contacting Claudia Kuehni by email: claudia.kuehni@ispm.unibe.ch.

## Supplementary Materials

The following are available online at https://www.mdpi.com/article/10.3390/vaccines9121496/s1. Table S1: Formulation of questions and answers from the special questionnaire on COVID-19 vaccinations sent to participants in May 2021. (COVID-PCD study, May 2021); Table S2: Characteristics of people with primary ciliary dyskinesia who completed the special questionnaire on COVID-19 vaccinations and those who did not. (COVID-PCD study, May 2021); Table S3: Demographic characteristics of adults with primary ciliary dyskinesia who were already vaccinated or willing to be compared to adults who were unwilling to get vaccinated. (COVID-PCD study, May 2021); Figure S1: Speed of vaccination uptake among adults in the COVID-PCD in countries with the highest number of participants. (COVID-PCD study, May 2021). Abbreviations: UK, United Kingdom; USA, United states of America; GER, Germany; IT, Italy; CH, Switzerland; FR, France; and AU, Australia. Figure S2: Cumulative percent of COVID-PCD study participants vaccinated (black thick line), compared with nationwide data from selected countries (coloured lines, data source: GitHub [45], accessesd on 24 August 2021).
