# Supplementary material for: COVID-19 Vaccinations: Perceptions and Behaviours in People with Primary Ciliary Dyskinesia
Source: Vaccines (Basel). 2021 Dec 17;9(12):1496. doi: 10.3390/vaccines9121496 (PMC8707304; doi:10.3390/vaccines9121496)
Supplement: Supplementary file 1 [file vaccines-09-01496-s001.zip › vaccines-1476894-supplementary.pdf]

Supplementary information

Supplementary Table S1: Formulation of questions and answers from the special questionnaire on COVID-19 vaccinations sent to participants in May 2021. (COVID-PCD study, May 2021).

Question	Answer category
Have you been vaccinated against COVID-19?	No<br>No, but I have an appointment to get vaccinated<br>Yes, I received one dose<br>Yes, I received two doses
Do you plan to get vaccinated against COVID-19?	No, I don't plan to get vaccinated<br>I am not sure if I want to get vaccinated<br>Yes, I plan to get vaccinated<br>Other (please explain)
Which COVID-19 vaccine did you get?	Pfizer-BioNTech (BNT162b2)<br>Moderna (mRNA-1273)<br>AstraZeneca, also called Oxford vaccine (AZD1222)<br>Janssen/Johnson & Johnson (Ad26.COV2.S.)<br>Sputnik V<br>CoronavAC (Sinovac)<br>BBIBP-CorV (Sinopharm)<br>EpiVacCorona<br>Convidicea (Ad5nCov) (CanSino Biologics)<br>Covaxin (Bharat Biotech)<br>Other<br>I don't know
What are your reasons to get vaccinated against COVID-19 (Please rate the reasons from unimportant to very important)	It will protect me from getting sick if I get infected with SARS-CoV-2<br>It will protect others from getting infected<br>It will help to stop the pandemic<br>It will allow me to see friends and family members again<br>It will allow me to travel abroad<br>It will allow me to go to the gym, cinemas, restaurants and other places<br>Side effects are usually mild<br>Other (please explain below)
What are your reasons for not wanting to get vaccinated against COVID-19 or want to wait and perhaps get vaccinated later? Please rate below how much you agree with the reasons below on a scale from strongly disagree to strongly agree.	I already had confirmed COVID-19 disease or tested positive for antibodies<br>I am concerned about side effects of the vaccine<br>I experienced side effects when getting the first shot of vaccine and now I don't want to get the second shot of vaccine<br>I want to see how the vaccine works on other people before I get it<br>Someone I know had a severe side effect after a COVID-19 vaccine and I am afraid this might also happen to me<br>I am concerned that the vaccine will affect my fertility<br>I am concerned that the development of the COVID-19 vaccines was too rushed<br>I am concerned about the ingredients in the vaccine<br>I don't think the COVID-19 vaccine will protect me<br>I would have to pay for it, and I can't afford it<br>I don't like needles<br>I think I would only get mild symptoms from COVID-19 if I got it<br>I am at low risk for getting COVID-19 so I don't need it<br>I would rather risk getting COVID-19 than the vaccine<br>I think that there will be enough other people in my community who will get the vaccine, to enable herd immunity, so I won't have to<br>Few people have COVID-19 in my area so I don't think I will get it<br>I don't like people telling me what I have to do<br>It's against my religious beliefs<br>I am concerned that the vaccine could give me COVID-19<br>COVID-19 is a hoax<br>I don't know where to get this vaccine<br>Other (please explain below)
Have you changed the way you protect yourself against COVID-19 since you	No, I shield the same way as before getting vaccinated by avoiding public places and seeing as few people as possible<br>Yes, now I go more often for grocery shopping<br>Yes, now I go more often for other shopping that groceries

got vaccinated? (e.g., you leave your house more often or you see more people)	Yes, now I go more often for appointments such as hairdresser and physiotherapy<br>Yes, now I go more often for exercise outdoors (e.g., walking, running, cycling)<br>Yes, now I go more often for exercise indoors (e.g., fitness, indoor team sports)<br>Yes now I go more often to my workplace<br>Yes, now I more often see family members<br>Yes, now I more often see friends<br>Yes, now I more often use public transport<br>Yes, I started shaking hands or hugging friends and family<br>Yes, I don't protect myself anymore because I am vaccinated<br>yes, other (please explain below)
If you have any comments about COVID-19 vaccines, please describe here	Freetext

Supplementary Table S2: Characteristics of people with primary ciliary dyskinesia who completed the special questionnaire on COVID-19 vaccinations and those who did not. (COVID-PCD study, May 2021).

	**Completed vaccination questionnaire**	**Did not complete vaccination questionnaire**
	N=423	N=266
	n (%)	n (%)
**Age, median (range)**	30 (1-85)	24 (1-69)
**Age groups**		
0-12 y	95 (22)	75 (28)
13-17 y	41 (10)	24 (9)
18 y or above	287 (68)	170 (63)
**Sex**		
Female	261 (62)	152 (57)
**Country of residence**		
United Kingdom	88 (21)	54 (20)
USA	64 (15)	59 (22)
Germany	76 (18)	19 (7)
Italy	24 (6)	22 (8)
Switzerland	31 (7)	16 (6)
France	23 (5)	16 (6)
Australia	14 (3)	13 (5)
Other European countries	81 (19)	36 (14)
Other non-European countries	22 (5)	31 (12)

Supplementary Figure S1: Speed of vaccination uptake among adults in the COVID-PCD in countries with the highest number of participants. (COVID-PCD study, May 2021). Abbreviations: UK: United Kingdom, USA: United states of America, GER: Germany, IT: Italy, CH: Switzerland, FR: France, AU: Australia.

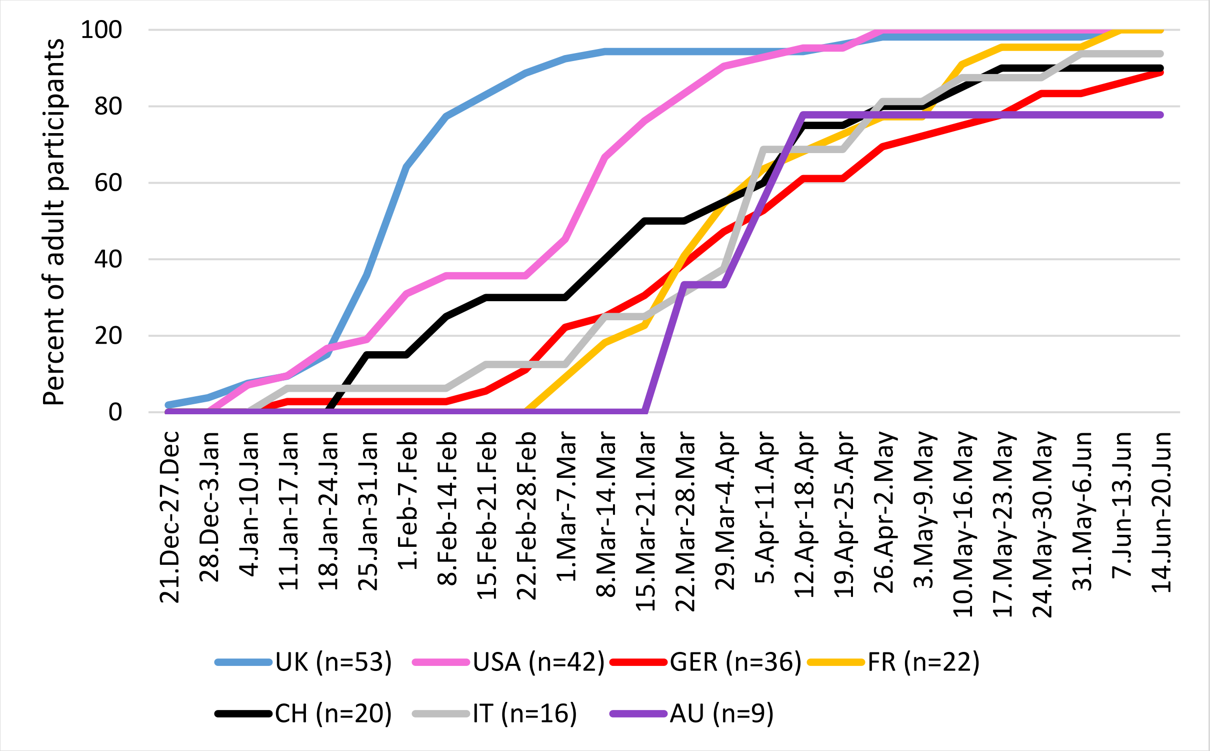

Supplementary Table S3: Demographic characteristics of adults with primary ciliary dyskinesia who were already vaccinated or willing to be compared to adults who were unwilling to get vaccinated. (COVID-PCD study, May 2021).

	**Vaccinated or willing to be**	**Unsure or unwilling to get vaccinated**	**p-Value**
	N=262	N=21	
	n (%)	n (%)	
**Age, median (range)**	41 (30-52)	37 (25-51)	0.210*
**Sex**			
Female	178 (69)	15 (71)	0.506#
**Country of residence**			0.023
United Kingdom	56 (21)	2 (10)	
USA	42 (16)	1 (5)	
Germany	37 (14)	5 (24)	
Italy	15 (6)	1 (5)	
Switzerland	16 (6)	4 (19)	
France	19 (7)	0	
Australia	6 (2)	3 (14)	
Other European countries	55 (21)	4 (19)	
Other non-European countries	16 (6)	1 (5)	

*Wilcoxon–Mann–Whitney test; #Fisher's exact.

Supplementary Figure S2: Cumulative percent of COVID-PCD study participants vaccinated (black thick line), compared with nationwide data from selected countries (colored lines, data source: https://github.com/owid/covid-19-data/tree/master/public/data/vaccinations/country_data, downloaded 24.08.2021).

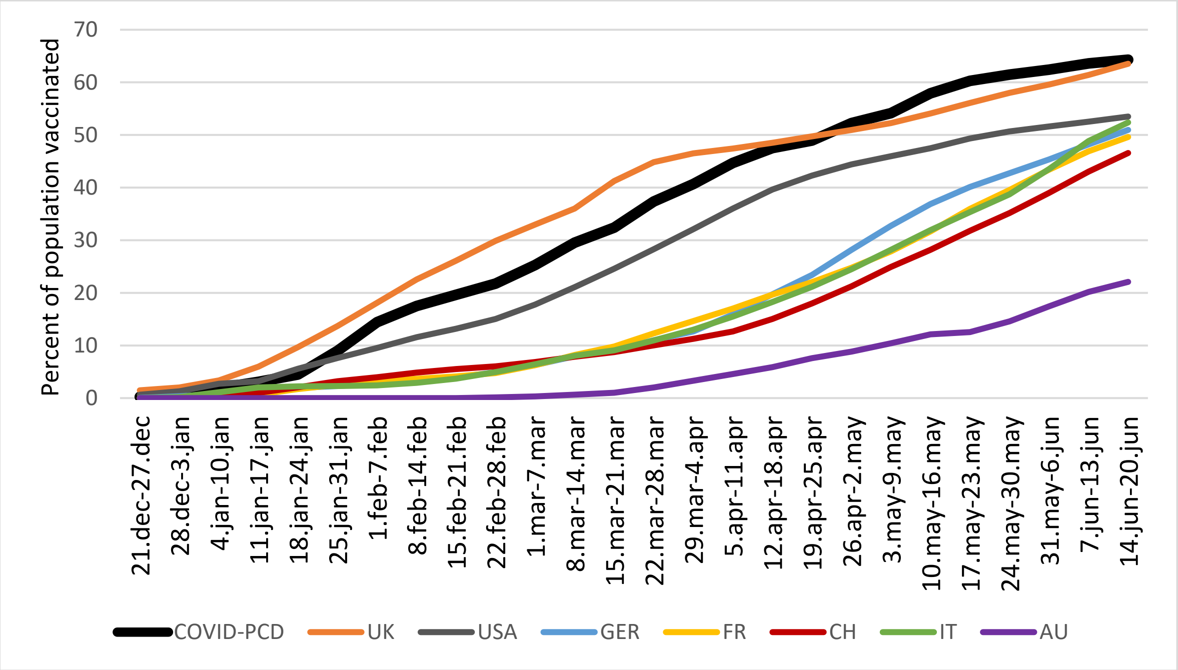

Nationwide data shown for countries from which at least 15 people participated in the COVID-PCD study by August 2021. Abbreviations: UK: United Kingdom, USA: United states of America, GER: Germany, IT: Italy, CH: Switzerland, FR: France, AU: Australia.
